# Cleaning up China’s Medical Cabinet—An Antibiotic Take-Back Programme to Reduce Household Antibiotic Storage for Unsupervised Use in Rural China: A Mixed-Methods Feasibility Study

**DOI:** 10.3390/antibiotics9050212

**Published:** 2020-04-27

**Authors:** Leesa Lin, Xiaomin Wang, Weiyi Wang, Xudong Zhou, James R. Hargreaves

**Affiliations:** 1 London School of Hygiene & Tropical Medicine, London WC1H 9SH, UK; leesa.lin@lshtm.ac.uk (L.L.); James.Hargreaves@lshtm.ac.uk (J.R.H.); 2 The Institute of Social and Family Medicine, School of Medicine, Zhejiang University, 866 Yuhangtang Rd., Hangzhou 310058, China; xiaominwang2018@zju.edu.cn (X.W.); weiyiwang@zju.edu.cn (W.W.)

**Keywords:** drug take-back, environment, community health, drug abuse, prescription drugs, antimicrobial resistance (AMR), RE-AIM, community-based participatory research (CBPR), feasibility, pilot

## Abstract

Background: Antibiotic misuse and unsafe disposal harm the environment and human health and contribute to the global threat of antimicrobial resistance. Household storage of antibiotics for unsupervised use and careless disposal of medications is a common practice in China and most low- and middle-income countries. Currently, few interventions are available to address this challenge. Objective: This study assesses the feasibility and acceptability of an evidence-based, theory-informed, community-based take-back programme for disposing household’s expired, unwanted, or unused antibiotics in rural China. Methods: We adopted the RE-AIM framework and the community-based participatory research principles in the development, implementation, and evaluation of the intervention. The RE-AIM (reach, effectiveness, adoption, implementation, and maintenance) and Medical Research Council’s frameworks were employed in analysing and reporting evaluation results. A mixed-methods, controlled pre-and post-test design was used for (1) quantitative surveying of a representative community panel of 50 households, and (2) qualitative semi-structured stakeholders’ interviews to explore intervention and study design feasibility and acceptability at three phases: pre-intervention, intervention, and post-intervention. Quantitative and qualitative data from a similar village—serving as a control—were also collected. Results: All a priori feasibility objectives were met: Conversion to consent was 100.0% (100 screened, approached, recruited, and consented). All participants completed the pre-intervention assessment, and 44/50 households in the intervention village completed the post-intervention assessment. The programme, embedded in existing social and physical infrastructure for dissemination, directly reached over 68.2% (30/44) of its target audience. Stakeholders reported the intervention and study design as feasible and acceptable. Conclusions: This study illustrates the feasibility, acceptability, and potential efficacy of community-based antibiotic take-back programmes in China to encourage safe disposal and decrease the availability of expired, unwanted, or unused antibiotics in the household for unsupervised use.

## 1. Introduction

The effectiveness of antibiotics has been undermined by decades of antibiotic misuse constituting a global health threat—antimicrobial resistance (AMR) [1,2]. In European countries, the burden of infections caused by antibiotic-resistant bacteria (170 DALYs per 100,000 population) is estimated to be equal to that of the combined burden of three major infectious diseases including influenza, tuberculosis, and HIV (183 DALYs per 100,000 population [3]. Comparatively, the prevalence of antibiotic resistant infections in low-and-middle income countries (LMIC) such as Brazil and Indonesia is predicted to rise 4–7 times faster than their European counterparts, leading to both economic and human cost [4,5]. A majority of human antibiotic consumption occurs in community settings outside of clinical facilities, especially in LMIC where antibiotic self-medication is close to 40%; half of these antibiotics come from household storage [6,7]. China, one of the world’s largest producers and consumers of antibiotics, faces among the most severe challenges of this crisis, with antibiotic residues and resistance genes detectable in surface water, waste water treatment plants, soil, vegetable produce, and animals [8,9,10,11]. Since 2011, the Chinese government has implemented a series of measures to contain this problem; however, most of these stewardship programmes focus on regulating prescriptions in hospitals and few address the easy access to antibiotics available in communities [12,13]. Nationwide surveys demonstrated that over 70% of Chinese households stored antibiotics that were eventually self-administered without professional supervision [6,14,15,16]. 

Recent reviews showed expired, unwanted, or unused (EUU) medicines were either stored unintentionally as leftovers or kept purposefully to treat similar conditions in the future (33%); among those who disposed of unused medicines, 50% used a take-back programme and 42% disposed the medicines in the trash or toilet [17,18,19,20]. The improper disposal of unused antibiotics can harm the health of the environment, wildlife, and humans, especially in countries, like China, with poor waste management systems [19]. The awareness and concern over the presence of pharmaceuticals in drinking water and the threat of misuse posed by EUU medications has led to interventions like ’Wise List’ of medicines for ambulatory care [21] and drug take-back programs for the removal of household access in developed countries (e.g., the United States, Sweden, and Germany) in the past decade [18,22]. Evaluations of take-back events demonstrated their positive effect on raising awareness about and reducing misuse or abuse of drugs [23,24,25,26]. “Ecopharmacovigilance” (EPV) has been an area of novel interest in Europe and North America for the past decade with an aim towards addressing issues associated with pharmaceuticals in the environment (e.g., water or soil) in a timely and appropriate way [27]. The attention on EPV in China is recent, and focuses on minimization of environmental risks posed by pharmaceutical residues and the need to guard against and control the pharmaceutical pollution source [28,29,30,31]. However, despite being one of the largest producers and consumers of antibiotics, discussions about safely disposing of antibiotics are practically non-existent in China. No interventions to date have attempted to address non-prescription household antibiotics use. There are few convenient and environmentally responsible disposal methods for systemically removing or reducing household antibiotic stockpiles in China, and public-targeted interventions are a pressing need. 

In this study, we employed a mixed-methods approach to develop and assess the feasibility and acceptance of an antibiotic take-back and disposal programme in rural China where antibiotic misuse in the community is the most severe [32,33].

## 2. Results 

### Feasibility and Acceptability of the Intervention

Table 1 reports the socio-demographic and background characteristics of the study participants. A total of 412 min of qualitative stakeholders interview data were collected (n = 21); each interview lasted approximately 10–34 min. 19 out of 21 respondents were female; all but three did not go to college. The mean age was 40.6 (±9.1) years.

**Figure 1:**
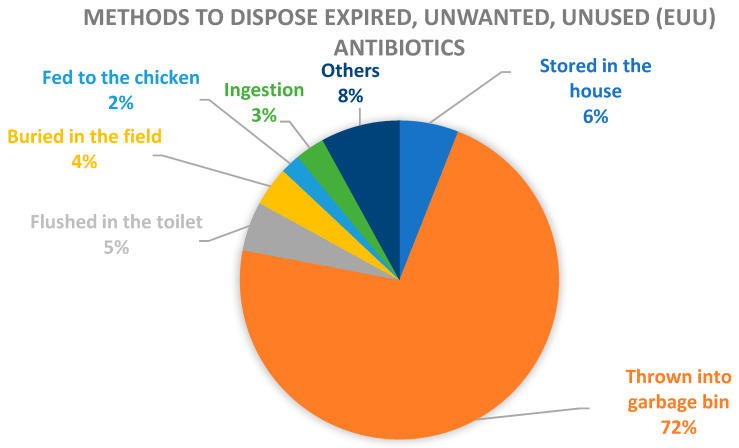
Methods to dispose expired, unwanted or unused (EUU) antibiotics.

The quantitative evaluation data are presented in Table 2 where 44 households in the intervention village and 39 households in the control village completed the post-intervention questionnaires with no missing data. Pre-intervention baseline assessment showed that the most common method to dispose expired, unwanted unused (EUU) antibiotics in the sampled villages was “thrown into garbage bin” (72%, 72/100), followed by “other methods” (8%, 8/100) and “stored in the house” (6%, 6/100), indicating the need for appropriate disposal methods (Figure 1). During the 30-day intervention period, roughly one third (7/22, 31.8%) of the households in the intervention village (who had self-reported having antibiotics stored at home prior to intervention) returned the antibiotics. Additionally, a month after the intervention, a follow-up assessment was conducted in pilot village to understand the change in awareness and perceptions of the potential danger of “non-prescription antibiotic use” and “unsafe disposal” on human and environmental health; 40 households in the intervention village completed the follow-up questionnaire with one household skipped several items (missing data). Due to the nature of the data and small sample size, these analyses are only useful for descriptive purposes.

Recruitment and retention: Fifty households in each study site were approached; all were eligible and recruited. The proportion of households approached who consented (conversion to consent) was 100%—well above the target set of 60.0%. Among them, 44 in the intervention village and 39 in the control village retained and completed the pre- and post-intervention questionnaires. 

Reach is measured by the percentage of residents who were informed about the programme and were potential users. Thirty out of forty-four households in the community panel had heard of the antibiotic take-back programme. A total of 13.3% had heard about it from WeChat and Women’s Federation, over 90% from print materials. 

Effectiveness is measured by participation in or intention to participate in the take-back programme. A total of 48 households used the bartering market (7 households from the community panel). Thirty-eight households intended to participate in the future and eight already recommended using the bartering market for antibiotic take-back to at least one other person in the past month.

Adoption: No barriers to adoption were identified by implementers. Not knowing about the take-back programme, no household storage, and no time to bring antibiotics in were listed as the top three reasons for non-participation by the community panel. Additionally, 34 households said they would recommend other villages to adopt the antibiotic take-back programme in their bartering markets.

Implementation of the programme, measured by *fidelity,* was delivered as intended. All eligible Women’s Federation members were actively involved in intervention delivery. A total of 48 households used the bartering market for antibiotic take-back and disposal; all returned antibiotics were properly sorted and documented according to study protocol, reported in Table 3. Intervention adherence and participant compliance was achieved.

Maintenance concerns the long-term maintenance of behaviour change at the individual level, which is not assessed in this study. At the village level, the potential for the antibiotic take-back programme to become a routine part of the culture is high. Among the 44 households who completed the post-intervention assessment, 40 interviewees thought the take-back programme should stay a part of the bartering market and be promoted to other villages, 4 stayed neutral, and none disagreed. 

Acceptability and appropriateness: The acceptability and appropriateness of the intervention is high. Awareness around the environmental protection is high. The intervention was appropriate, acceptable and sustainable to the implementers, the Women’s Federation. Additionally, among the four households in the control village that had heard of the take-back programme, two reported intention to participate, which will indicate *scalability*.

Process evaluation outcomes are reported in Table 3. In brief, a total of 50 boxes of antibiotics were collected during the first 30 days of the programme, valued at 592 RMB worth of household items at the bartering market—an average of 11.84 RMB per box. Health education messages were disseminated via WeChat (no cost), SMS and posters and pamphlets (approximately 1200.00 RMB). 34 respondents mentioned “to protect the environment” as the main reason to continue or to expand the bartering market for antibiotic-take back, 18 “to prevent inappropriate use at home”, and 12 “because there is no other platform to safely dispose antibiotics", while 10 respondents felt “incentivized by the household items at the bartering market”.

Data that address 14 methodological issues of feasibility research for full-scale intervention development are presented in Table 4.

## 3. Discussion

This study presents the high feasibility and acceptability of a community-based antibiotic take-back service offered at a local bartering market for recyclables. The overall positive feedback supports the need and warrants the continuation and expansion of the programme. There is a lack of environmentally safe disposal guidelines and take-back services for the proper disposal of antibiotics in China. This proposed intervention served a dual-purpose: (a) To reduce access to unnecessary antibiotics in the household and the likelihood of self-medication with antibiotics without supervision, and (b) to promote safe disposal and protect the environment. Villagers confirmed the existing local town-run bartering market for recyclables as a convenient site for an antibiotic disposal programme. These bartering markets are often located in village centres accessible to most residents and managed by local officials or members of local Women Federation chapters, well-known to the community. As such, the costs (approximately 1800.00 RMB) were minimal when the proposed intervention was well-embedded in existing infrastructure, which further improved feasibility and scalability. Our data showed removal of household antibiotic storage can reduce the likelihood of self-medication with antibiotics. 

Strengths of this study include utilisation of a mixed-methods approach and adoption of the RE-AIM and MRC evaluation frameworks to achieve the study aims. Also, by following the community-based participatory research (CBPR) principles, this project had two distinctive features: *Local ownership* and a *realist approach* throughout the adaptation and implementation process. First, the community partners were involved in plans and development from the beginning and had real influence on the project; their involvement helped to develop the understanding of the “contexts” and “mechanisms” that constitute the effectiveness of behavioural change interventions. Second, with RE-AIM constructs embedded in the study design since project inception, we were equipped to identify “what works for whom, in what contexts, and how”. The findings from this study should be interpreted with several limitations. The small sample and use of one site may seem to limit the results’ generalisability. Because data were collected from a representative sample of rural Chinese residents in the participating site, representing 5.5% (50/916) of the households, and from a control site (11.2%, 50/447) at three different time points, the general pattern of findings observed in this study is sufficiently robust for a feasibility study to alleviate concerns about potential spuriousness. This investigation offers needed empirical feasibility data on the antibiotic take-back programme for a large trial.

### Interpretation of Findings

This study identified a critical gap of current AMR strategy in the Chinese infrastructure where EUU antibiotics in the community are left unattended. There is a lack of knowledge of and platform for proper disposal and a strong interest in participating in take-back programmes. Formative data found that the local awareness and concern over the presence of pharmaceuticals in drinking water and the threat of misuse were high in both intervention and control villages, yet self-medication with antibiotics were common among local residents who seemed to be unsure of what constitutes proper disposal and showed reluctance in giving up habits of household storage of antibiotics. We found individual’s health decisions about antibiotic use to be complex and not entirely driven by their cognitive and rational characteristics—contextual factors, including access to antibiotics and interpersonal connections, are equally or more critical to healthcare decision-making processes. Evidence showed when information or time is limited and complexity of the situation is overwhelming, individuals often combine rationality with other sources of so-called tacit or experiential knowledge and utilise strategies such as trust, intuition, and emotion to assist decision making [34]. Antibiotic misuse in China is driven by a complex set of factors embedded in its culture and beliefs, health system, and society [13,16,35]. Data from this project highlighted that increasing knowledge and raising awareness about the consequences of the inappropriate use and disposal alone is unlikely to enable the desired behaviour change. A complex intervention that also supports prudent prescriptions, reduces over-the-counter purchases, and improves dispensing systems to reduce leftover prescriptions in addition to the proposed community-based intervention will be necessary. Further clarifications about what constitutes “appropriate practices” in the given context should be included in the education intervention. In our sampled villages, respondents who engaged in misuse behaviours such as feeding children with antibiotics, burying them in the field, taking them before expiration, or not thinking antibiotics can “go bad” might consider their behaviour as “being completely appropriate”. Changing the local social and infrastructure environments for appropriate antibiotic use and disposal while providing actionable information about how and when/where to use and dispose antibiotics are key to cue people to action. Educating about how to care for common self-limiting illnesses and non-antibiotic alternatives for symptom relief will improve the likelihood for better use of antibiotics. Health education messages for the project should address these concerns during full scale implementation. This study also informed sampling and data collection strategy during full scale implementation. We found that many younger adults of a working age stayed away during the week for work, leaving only grandparents and children in the village; it was therefore best to reach them over weekends. This scenario has important implications on the planning of data collection when large sample size is involved as it restricts the number of days allowed for data collection. Furthermore, it is concerning that within 30 days, we saw a sharp decrease in the household antibiotic storage in the pilot village from 34.0% to 27.8% in the absence of an intervention. There might be several possible explanations for this phenomenon: for example, a Hawthorne effect (also referred to as the observer effect) in which individuals modify their habits of storing antibiotic at home in response to their awareness of being observed. However, we ruled out this possibility because this effect was not seen in the intervention village which was also being observed. Also, formative data suggested that unlike prescription drug diversion in the U.S. which might be viewed as a type of behavioural disorder carrying a potential social stigma [36], in China keeping antibiotics at home for future use is a socially acceptable common practice [37,38]. The concern over under-reporting of household storage of antibiotics is low. Furthermore, the quantity of household storage of antibiotics was verified by an inspection of the household medical cabinet onsite, leaving little room for error in reporting. A small sample size, a short study duration, or the timing of data collection (e.g., flu season or not) may also be variables in play. However, this speculation could not fully explain the sudden drop in the storage observed in the control village, which calls for further qualitative investigation. On the other hand, since there is currently no mechanism in place to remove the excess antibiotics from these households, the reduction in the storage can only be assumed to either have been consumed without a prescription or discarded inappropriately. This discovery was worrisome, especially considering the timing of the feasibility study (June) was not peak season for upper respiratory tract infections (URTIs) and was low season for antibiotic consumption. Given this timing, compounded with easy access to antibiotics and the population size of 577 million rural residents, it is clear that the severity of misuse and mishandling of antibiotics in the community requires an urgent need for intervention. Nevertheless, during the 30-day period, this programme was able to reach a sizable portion (68.2%, 30/44) of the intended target audience with messages promoting the safe disposal of antibiotics, and among them, 26.7% (8/30) further spread this message, including people outside of the intervention villages. 

The frequent exchange of information between villages reported in this study also indicated that in a full-scale study, township-level randomisation—rather than village-level—would be appropriate as part of a cluster trial. Choice of sampling approach (e.g., “fried-egg” design [39]—sampling individuals from villages centrally located within each township) will need to be handled with care to prevent “contamination” between intervention and control groups. Future research on social networks may be able to generate additional insight regarding the diffusion of innovations for reducing antibiotic misuse. Moreover, given the high levels of antibiotic residues in fresh water and soil in China, future studies should explore whether those more conscious about environmental protection are more likely to engage in prudent antibiotic use and disposal, which may inform a “One Health” approach. Finally, we recognise that although the proposed intervention will remove household antibiotic stockpiling, it will not address all the challenges associated with antibiotic misuse in the community. For example, community pharmacies have been identified as the main source of antibiotics for self-medication in China [7] despite a national policy that bans non-prescription sale of antibiotics. Future intervention could include pharmacists as antibiotic stewards [40,41] and train them to better advise consumers on safe disposal of EUU antibiotics and the responsible management of self-limiting URTIs such as offering alternative symptom relief options for the common cold or flu. A multifaceted complex intervention that also enforces regulations regarding the sale of antibiotics and pack-based antibiotic dispensing systems to reduce leftover antibiotic prescriptions is necessary to curb the main sources of non-prescription antibiotics for self-medication use. 

This study filled the knowledge gap by describing systematic steps taken to adapt community-based interventions for a new context and a new health risk. From a global health perspective, the results of this study demonstrate that a take-back programme can be a potentially effective instrument for decreasing the availability of unnecessary antibiotics and potential misuse in communities across China and around the world, especially in low-and-middle income countries. As most rural Chinese towns have bartering markets, the proposed intervention has great potential for significance and scalability. 

## 4. Methods

This study aims to determine the feasibility and acceptability of the proposed intervention, an antibiotic take-back programme in rural China. The proposed intervention consists of two components: A community-based antibiotic take-back programme (embedded in the existing “bartering market” for recyclables in rural Zhejiang province) and health education. We first pre-tested intervention materials and implementation methods with experts and potential users for validity and appropriateness. Second, we explored stakeholders’ views on potential facilitators and barriers to the intervention. Last, utilising a mixed-methods design, we assessed the feasibility, acceptability, and scalability of a pilot intervention and explored its effectiveness. The study design and process of adapting existing interventions to new populations and settings are reported in detail elsewhere.

### 4.1. Feasibility Study Design

Guided by the Medical Research Council’s (MRC) framework for the development and evaluation of complex interventions [42] and the RE-AIM (reach, effectiveness, adoption, implementation, and maintenance) framework [43], this mixed-methods research design comprised a controlled pre–post quantitative component and embedded qualitative component. The study methodology’s feasibility was first examined using the following quantitative data: Recruitment, retention, follow-up measure response rates, missing follow-up measure data, and usage data. The study design and intervention’s feasibility and acceptability were then explored using qualitative semi-structured interviews with stakeholders. We noted that this pilot study was designed to test the feasibility of one component of a large community-based complex intervention, not the efficacy or effectiveness of the new intervention, which is the aim of a full-scale randomized controlled trial (RCT) [44]. Lastly, we systematically explored and addressed the 14 potential methodological issues of feasibility studies identified by Bugge et al. and Shanyinde et al. [45,46].

### 4.2. Setting and Sample 

Feasibility data for the intervention came from a representative community panel of 100 households in two rural villages—one intervention and one control—in Zhejiang, China, conducted over the first 30 days of implementation of an antibiotic take-back programme in June 2019. All households in the villages were eligible for inclusion and those agreeing to participate gave informed consent. Due to the intervention design and the local context, we targeted the self-identified female heads of household. Qualitative data came from 21 purposively-selected stakeholders of the community, who represented the characteristics relevant to the study setting in terms of age, gender, socio-economic status, and community roles. 

### 4.3. Data Collection and Management

For pre- and post-intervention evaluations with the community panel, face-to-face household surveys consisted of quantitative and qualitative items assessing antibiotic use and disposal behaviours, exposure to and participation in the programme, and public knowledge and perceptions about antibiotic use. Inspections of household medical cabinets were conducted at the end of each survey. Stakeholders, including residents, local government officials, community partners, potential implementers of the intervention, community pharmacies and clinicians, and local residents, were recruited for semi-structured interviews and to access process evaluation data in the pilot village. Pre- and post-intervention evaluation data—both quantitative and qualitative—were also collected from the control village with a similar sample. Stakeholders’ interviews were audio-recorded, transcribed by an independent transcription company, checked for accuracy, anonymised and imported into Nvivo11 software to facilitate qualitative data analysis according to RE-AIM framework.

### 4.4. Sample Size

While a sample size was not calculated (outcomes of interest were intervention and study design feasibility and acceptability), previous studies have identified a minimum of 20 participants is required to identify 95% of usability problems [47]. Although there is currently no published guidance as to the sample size required for a pilot or feasibility trial and given that this pilot study employed a controlled pre-and-post design (not a trial), we set the sample size to be 50 households per arm, which was higher than the median among the published UK pilot trials [48]. This intervention was delivered at the village rather than the individual level. In a full-scale study, village- or township-level randomisation as part of a cluster trial would be appropriate. For this study, the feasibility of randomisation was not tested. 

### 4.5. Measures

The intervention aimed to reduce household antibiotic storage and improve safe antibiotic disposal; this informed measure selection. The feasibility and acceptability of the selected study measures were assessed to determine those most appropriate for a future cluster trial. 

Primary measures: The primary objective was to describe antibiotic storage and disposal behaviours. All respondents were asked whether, in the past month, they have: (a) Kept antibiotics at home and (b) participated in the take-back programme. 

Secondary measures included awareness and perceptions of the potential danger of “unsafe disposal” and “non-prescription antibiotic use” on human and environmental health.

Process evaluation: Routine data on programme utilisation, costs, and in-kind expenses were calculated. Returned antibiotics were stored in a pre-prepared bag with a pre-designed information sheet including details of each collection, e.g., types and amount of the drugs received, and user’s satisfaction. 

Data on participants’ socio-demographic characteristics including sex, age, education, income, employment, and number of children in the household were also collected.

### 4.6. Data Analysis

Descriptive statistics (frequencies, means and standard deviations) were calculated for all variables. Qualitative data were analysed using framework analysis. A priori codes were drawn from the interview topic guide, study objectives, and feasibility evaluation framework. LL was the primary coder and interpreted the data, along with two other coders, WXM and WWY. Consensus on themes and key findings were reached through discussion.

## 5. Conclusions

This feasibility study presents an overall favourable public response toward a theory-driven, community-based bartering market for antibiotic-take-back as a feasible, acceptable, and appropriate intervention, warranting the expansion of the pilot programme. The proposed public-target intervention can be an important component of a multifaceted AMR strategy to decrease inappropriate antibiotic use in the community, especially in low-and-middle income countries, including China. 

## Figures and Tables

**Table 1:** Sample characteristics.

	Intervention		Control
**Population Size**	3015		1624
**No. of Household**	916		447
**Sample Size**	**Baseline Survey 50 (n,%)**	**Stakeholders Interview 21 (n,%)**	**Baseline Survey 50 (n,%)**
Sex			
Woman	42 (84.0)	19 (90.5)	36 (72.0)
Man	8 (16.0)	2 (9.5)	14 (28.0)
Age			
Minimum	23	24	22
Mean (SD)	45.8 (10.0)	40.6 (9.1)	49.1 (15.2)
Maximum	65	54	72
Highest Attainment Education			
College or above	3 (6.0)	3 (14.3)	7 (14.0)
High school	11 (22.0)	5 (23.8)	10 (20.0)
Middle school	24 (48.0)	10 (47.6)	17 (34.0)
Primary school or less	12 (24.0)	3 (14.3)	16 (32.0)
Income			
>10,000	3 (6.0)	0	8 (16.0)
5001–10,000	16 (32.0)	6 (28.6)	9 (18.0)
3001–5000	17 (34.0)	12 (57.1)	16 (32.0)
<3000	14 (28.0)	3 (14.3)	17 (34.0)
Employment			
Yes	21 (42.0)	9 (42.3)	11 (22.0)
No	29 (58.0)	12 (57.1)	39 (78.0)
Children in the household			
Yes	47 (94.0)	19 (90.5)	33 (66.0)
No	3 (6.0)	2 (9.5)	17 (34.0)
Having an active WeChat account			
Yes	40 (80.0)		32 (64.0)
No	10 (20.0)		18 (36.0)
How often do you use WeChat?			
All the time	27 (67.50)		27 (87.38)
Frequent	9 (22.50)		2 (6.25)
Sometimes	2 (5.0)		1 (3.13)
Not frequent	1 (2.50)		2 (6.25)
Never	1 (2.50)		0 (0.0)
Do you participate in the waste sort and recycle initiatives?			
Yes	41 (82.0)		39 (78.0)
No	9 (18.0)		11 (22.0)
Have you ever used the bartering market for recyclables?			
Yes	11 (22.0)		4 (8.0)
No	39 (78.0)		46 (92.0)

**Table 2:** Awareness of the danger of antibiotic resistance and unsafe disposal and associated practices among community panels.

Intervention Components	Intervention Village N (%)			Control Village N (%)	
	PRE-	POST-	FOLLOW UP *	PRE-	POST- *
	N = 50	N = 44	N = 40	N = 50	N = 39
**Health education strategy** **Knowledge and attitudes toward self-medication with and disposal of antibiotics**					
Antibiotic overuse may increase antibiotic resistance					
Agree	33 (66.0)	35 (79.5)	30 (75.0)	37 (74.0)	27 (71.1)
Neutral	11 (22.0)	6 (13.6)	9 (22.5)	12 (24.0)	8 (21.1)
Disagree	6 (12.0)	3 (6.8)	1 (2.5)	1 (2.0)	3 (7.9)
Inappropriate disposal of antibiotics can harm the environment					
Agree	45 (90.0)	42 (95.4)	37 (92.5)	40 (80.0)	31 (81.6)
Neutral	4 (8.0)	2 (4.6)	2 (5.0)	5 (10.0)	6 (15.8)
Disagree	1 (2.0)	0	1 (2.5)	5 (10.0)	1 (2.63)
Inappropriate disposal of antibiotics can harm the environment, I will dispose it appropriately					
Agree	44 (88.0)	40 (90.9)	37 (92.5)	35 (70.0)	35 (89.8)
Neutral	10 (5.0)	4 (9.1)	2 (5.0)	10 (20.0)	2 (5.1)
Disagree	1 (2.0)	0	1 (2.5)	5 (10.0)	2 (5.1)
Inappropriate disposal of antibiotics can harm the environment, I know how to dispose it appropriately					
Agree	29 (58.0)	28 (63.6)	27 (67.5)	21 (42.0)	20 (51.3)
Neutral	13 (26.0)	6 (13.6)	5 (12.5)	13 (26.0)	6 (15.4)
Disagree	8 (16.0)	10 (22.7)	8 (20.0)	16 (32.0)	13 (33.3)
Self-medication with antibiotics might have an adverse impact on our health					
Agree	41 (82.0)	44 (100.0)	34 (85.0)	44 (88.0)	32 (84.2)
Neutral	7 (14.0)	0	5 (12.5)	3 (6.0)	4 (10.5)
Disagree	2 (4.0)	0	1 (2.5)	3 (6.0)	2 (5.3)
Self-medication with antibiotics might have an adverse impact on health, one should not take antibiotics without professional supervision					
Agree	42 (84.0)	42 (95.6)	31 (79.5)	38 (76.0)	29 (74.4)
Neutral	4 (8.0)	0	4 (10.3)	8 (16.0)	5 (12.8)
Disagree	4 (8.0)	2 (4.6)	4 (10.3)	4 (8.0)	5 (12.8)
Self-medication with antibiotics might have an adverse impact on our health, one should not store antibiotics at home					
Agree	24 (48.0)	30 (68.18)	18 (46.1)	26 (52.0)	24 (63.2)
Neutral	12 (24.0)	6 (13.6)	7 (18.0)	12 (24.0)	10 (26.3)
Disagree	14 (28.0)	8 (18.2)	14 (35.9)	12 (24.0)	4 (10.5)
Participation in the antibiotic take-back programme					
Household antibiotic storage at the time of survey					
Yes	25 (50.0)	22 (50.0)	18 (45.0)	17 (34.0)	8 (21.1)
No	25 (50.0)	22 (50.0)	22 (55.0)	33 (66.0)	30 (78.9)
Participation in the take-back programme					
Yes	-	7 (31.8)	6 (33.3)	-	-
No	-	15 (68.2)	12 (66.7)	-	-

* Some items had missing data from one household. - no participation data collected from pre-intervention assessment and the control group.

**Table 3:** Process evaluation on the antibiotic take-back programme.

**Quantitative Data**			
**Health Education Strategy**			
No. of households in the intervention village completed post-evaluation		44 households	
No. of households received the health education messages		30/44 households (68.2%)	
No. of households further spread this message		8/30 households (26.7%)	
**Bartering Market for Household Expired, Unwanted, or Unused (EUU) Antibiotics**			
No. of households participated in the bartering market (including those who are not in the community panel)		48 households	
Antibiotics take-back via the bartering market		No. of box	
Cephalosporin (cefaclor, ceftriaxone sodium) Penicillin (amoxicillin) Quinolones (norfloxacin, ofloxacin) Macrolides (Azithromycin) Nitroimidazoles (Tixiaozuo) Others (non-antimicrobials/non-antibiotics)		10 11 2 7 1 19	
Total no. of returned antibiotics (boxes)/total costs		50 boxes/RMB 592	
**Qualitative Data: Users’ Opinions on the Feasibility of the Bartering Market.**			
	**Participants**		**Non-Participants**
**Acceptability of the bartering market**	**1. I have seen health education materials and realized that overuse of antibiotics can cause harm to the human body.** *“It is written on the leaflet that it is not good to take too much of it, so I brought it here.”* (Male, 65 years old, primary school) *“In the past, I would put some medicine at home, and I would take it when I subsequently got sick. I think the doctors actually prescribe more or less the same medication, but after reading the leaflet, I felt these materials are very useful. It is bad to take too much of it, and you can’t do this either. It has to be placed at the recycling point.”* (Female, 42 years old, high school graduate)		**1. I saw the relevant materials but was too late to take them to the bartering market.** *“Recently, it was really busy at home. I didn’t have time to take it there. In the future, if I have time here, I will take it there. It [the bartering market] is just a stone’s throw away, so it is very convenient.”* (Male, 48 years old, high school graduate)
	**2. Throwing antibiotics anywhere can pollute the environment. They are better handled by the bartering market.** *“The medicine is left at home, and it will be thrown away after a long period time. [I learned that] It will pollute the environment, so I brought it to the bartering market after seeing the ad.”* (Male, 65 years old, primary school graduate) *“It is not good to throw medicine as one pleases. You can’t throw them away randomly. After reading the text messages carefully, I felt there was something to gain.”* (Male, 62 years old, middle school graduate) *“I saw a notice saying that throwing medicine along with other garbage would pollute the environment. The bartering market is very good and can be taken advantage of.”* (Female, 40 years old, middle school graduate)		**2. There is no reserve of antibiotics at home.** *“We are usually in Wenzhou; there are no antibiotics at home. I don’t really like keeping too much medicine at home.”* (Female, 29 years old, high school graduate) *“We have no medicine at home, but after reading this material, I will be willing to take it there in the future.”* (Male, 43 years old, high school graduate)
	**3. There is no use keeping it at home. There are even gifts redeemable at the bartering market.** *“It is useless for me to keep medicine at home. The bartering market is quite good, and there are even redeemable gifts there, so they can be taken advantage of.”* (Female, 40 years old, middle school graduate)		**3. No relevant health education materials were received.** *“I didn’t receive the text messages. It may be that there was something wrong with the mobile phone. We are already old, so we don’t always check our mobile phones. I don’t know where the leaflet was placed; it could no longer be found.”* (Female, 49 years old, middle school graduate)
	**4. I don’t know how to handle it correctly myself.** *“I seemed to have set it up for a period of time before, but no one put it there. We usually just keep it at home, and I am worried that the children will take it randomly. If there is a recycling point, it will be more convenient because one can just put it directly there. Directly throwing antibiotics into an ordinary trash can doesn’t seem too good either, but we don’t know how to deal with it.”* (Female, 33, high school graduate)		**4. If something remains, I can use it next time. I am not very willing to take it there.** *“I also know that if it is just a small illness, one just needs to rest a few days even without taking medication to get well. But when one goes to work, they cannot rest for several days. I have to keep the medicine for use in the future. I don’t want to buy medicine again. The symptoms are similar every time. And the medicine prescribed by the doctor is more or less the same. Just taking the same medicine as last time is enough; taking medicine makes one recover faster. And some medicines have one or two left, and I would be embarrassed to take them there in exchange for a gift.”* (Male, 31 years old, college graduate)
**Acceptability of the Incentives**	*“I think that ordinary soap, scented soap, toothpaste and other similar things can be used, it would be very good, I personally like it.”* (Female, 42 years old, high school graduate) *“As regards gifts, it’s hard to say. Personal needs are different, and more choices are better.”* (Female, 40 years old, middle school graduate)		*“Some medicines have one or two pieces left, and I would be embarrassed to take them there in exchange for a gift.”* (Male, 31 years old, college graduate)

**Table 4:** Summary of the findings against 14 methodological issues for feasibility research.

Methodological Issues	Findings	Evidence
**1. Did the feasibility study allow a sample size calculation for the main trial?**	Yes	50 household approached 50 households eligible 50 households consent to participate in the study 48 households used the bartering market; 7 households were from the panel
**2. What factors influenced eligibility and what proportion of those approached were eligible?**	All households were eligible	All households were eligible
**3. Was recruitment successful?**	Yes	50/50 (100%) households agreed to participate in the panel
**4. Did eligible participants consent?**	Good conversion to consent	Fifty recruited out of 50 eligible, consent rate of 100.0%
**5. Were participants successfully randomised and did randomisation yield equality in groups?**	Not applicable in this study	Not applicable in this study
**6. Were blinding procedures adequate?**	Not applicable in this study	Not applicable in this study
**7. Did participants adhere to the intervention?**	Good adherence to the protocol	All take-back antibiotics were returned and documented according to the protocol.
**8. Was the intervention acceptable to the participants?**	acceptability explored in qualitative interviews	Residents from the intervention and control sites and the implementers found the intervention acceptable
**9. Was it possible to calculate intervention costs and duration?**	Yes	Costs for resource utilisation were assessed for participant use of antibiotic take-back programme and in-kind wage of implementors
**10. Were outcome assessments completed?**	There was no missing data from the take-back bartering market or from the household surveys.	There was no missing data as outcome data were collected in person.
**11. Were the outcomes measured the most appropriate outcomes?**	Outcome measures used did assess main outcomes of interest	Bartering market use data, household antibiotic stocks, and returned antibiotic were documented and analysed.
**12. Was retention to the study good?**	Good (88.0)	Response rates: Pre-intervention assessment (50/50) Post-intervention assessment (44/50)
**13. Were the logistics of running a cluster randomised controlled trial addressed?**	The buy-in from the Women’s Federation on site positively influenced the logistical running of study	There were no difficulties identified in the various processes and the researcher’s ability to implement them. Residents once recruited were readily identified.
**14. Did all components of the protocol work together?**	There were no difficulties identified in the various processes and the researcher’s ability to implement them.	Residents and the implementer (i.e. the Women’s Federation) found the intervention acceptable, feasible, and easy to implement.

## Data Availability

The datasets generated and/or analysed during the current study are not publicly available due to the likelihood of compromising the privacy of participating individuals given the small population size in the project sites; the data are available from the corresponding author upon reasonable request.

## Abbreviations

AMR: antimicrobial resistance; CBPR: community-based participatory research; EPV: ecopharmacovigilance; EUU: expired, unwanted, or unused; LMIC: low-and-middle-income countries; MRC: Medical Research Council; RE-AIM reach, effectiveness, adoption, implementation, and maintenance; RCT: randomized controlled trial; URTIs: upper respiratory tract infections.
